# Optical Coherence Tomography for Detection of Dental Cracks and Vertical Root Fracture: A Scoping Review

**DOI:** 10.1002/cre2.70323

**Published:** 2026-03-09

**Authors:** MHD. Mouaffak Alkhani, Ahmad Yasser Albittar, Uzma Munawwar Shaikh, Muhammad Takriti, Aylin Baysan

**Affiliations:** ^1^ Department of Oral Research, School of Dentistry University of Alabama at Birmingham Birmingham Alabama USA; ^2^ Department of Advanced Education in General Dentistry, School of Dentistry Columbia University New York New York USA; ^3^ Jacksonville University School of Orthodontics Jacksonville Florida USA; ^4^ Maharashtra University of Health Sciences Nashik India; ^5^ Department of Periodontics, School of Dentistry University of Illinois Chicago Chicago Illinois USA; ^6^ Centre for Oral Bioengineering, Barts and the London Faculty of Medicine and Dentistry Queen Mary University of London London UK

**Keywords:** apical periodontitis, diagnostic tools, endodontic disease, optical coherence tomography, periodontal disease, vertical root fracture

## Abstract

**Objectives:**

Vertical root fractures (VRFs) pose significant clinical challenges and may result in tooth loss. Current diagnostic methods, including conventional radiography and CBCT, are challenging to detect VRFs, especially in the early stages. Optical coherence tomography (OCT) has recently been introduced as a non‐invasive imaging technique for dental diagnostics. However, current knowledge of OCT in endodontics requires further research. This Article will review current literature on OCT and evaluate its potential as a diagnostic tool for VRFs in endodontics.

**Methods:**

A comprehensive scoping review was conducted using electronic databases including PubMed, Scopus, Ebsco, Google Scholars, and Embase without any language restrictions up to January 2025, focusing on OCT applications in diagnosing cracks and VRFs. Google and Open‐Grey were used to search for grey literature alongside handsearching. Clinical and laboratory‐based studies conducted on adult human‐teeth were considered eligible. A total of 28,303 studies were generated when screened in the last 20 years; 27,887 studies were found relevant, and 416 duplicates were removed. Following title and abstract screening, a total of 36 studies were considered for full‐text review. Ten studies met the inclusion criteria and were included in this review.

**Results:**

OCT system demonstrated high specificity (63%–100%) and sensitivity (83%–98%) in detecting cracks and VRFs. SS‐OCT demonstrated promising imaging capabilities and deep penetration. OCT offers advantages over radiographs and CBCT in non‐radiation exposure and real‐time imaging. OCT systems could be considered in clinical practice following improvements in relation to penetration depth and intra‐oral adaptation.

**Conclusions:**

Within the limitation of this scoping review, the use of OCT is promising for detection of cracks and VRFs. By addressing the limitations related to penetration depth, mechanical design, and soft tissue imaging, OCT may find its path into clinical adoption, including endodontics. Areas that can help includes developing OCT systems tailored for routine endodontic use and assessing long‐term impacts in the field.

## 1 Introduction

A tooth crack is a fracture plane of unknown depth and direction passing through the tooth structure that may progress to communicate with the dental pulp and/or periodontal ligament (Ellis 2001). The American Association of Endodontists classifies longitudinal tooth fractures into five distinct types based on their location, extent, and clinical implications (Rivera and Walton 2008). Craze lines are superficial cracks that affect only the enamel and are typically asymptomatic, posing no risk beyond aesthetics (Ellis 2001; Rivera and Walton 2008; Tsesis et al. 2010; Haupt et al. 2023). Fractured cusps involve a complete or incomplete break starting from the crown and extending subgingivally, typically affecting one cusp and often associated with large restorations (Ellis 2001; Rivera and Walton 2008; Tsesis et al. 2010; Haupt et al. 2023). A cracked tooth features an incomplete fracture initiated from the crown that extends subgingivally, typically mesiodistally, potentially involving both marginal ridges and progressing apically, all of which are challenging to diagnose and treat (Ellis 2001; Rivera and Walton 2008; Tsesis et al. 2010; Haupt et al. 2023). Split teeth is an advanced stage of a cracked tooth, where the fracture extends completely from the crown to the root, separating the tooth into distinct segments; treatment options are limited, often requiring extraction or removal of the smaller fragment with possible retention of the remaining tooth (Ellis 2001; Rivera and Walton 2008; Tsesis et al. 2010; Haupt et al. 2023). The final form of cracks and the most notable in endodontics is the vertical root fractures (VRFs). VRFs are forms of cracks, characterized by cracks extending between the root and crown that result in tooth loss (Ellis 2001; Rivera and Walton 2008; Tsesis et al. 2010; Haupt et al. 2023). This presents significant diagnostic and therapeutic challenges in Clinical Endodontics due to their subtle clinical presentations and multi‐factorial etiology (Haupt et al. 2023; Torabinejad et al. 2020). According to published cases related to VRFs, fractures are notably prevalent in endodontically treated teeth subjected to excessive removal of tooth structure, potential undesirable force implications during root canal obturation, posts, and/or post‐space preparation.

Age and gender influence the VRF incidence. VRF's rates are 78.5% high in patients over 40, with a peak at 37% in ages 40–49, followed by ages 50–60 at 28.13% (Pack 1994; Nicopoulou‐Karayianni et al. 1997; Cohen et al. 2006; Wilcox et al. 1997; Chan et al. 1999). Some studies noted high prevalence in females (67.35%:32.65%) in endodontically treated teeth (Torabinejad et al. 2020; Nicopoulou‐Karayianni et al. 1997; Chan et al. 1999; Khasnis et al. 2014; Lee et al. 2023). Interestingly, epidemiological studies reported that 10.9% of dental extractions are due to VRFs, with maxillary second premolars and mandibular molars being the most affected (Cohen et al. 2006; Tamse et al. 1999; Talwar et al. 2016). The prevalence of VRFs in extracted endodontically treated teeth ranges from 2% to 20%, highlighting significant clinical variability (Lee et al. 2023; Rathke et al. 2022; Mireku et al. 2010).

**Figure 1:**
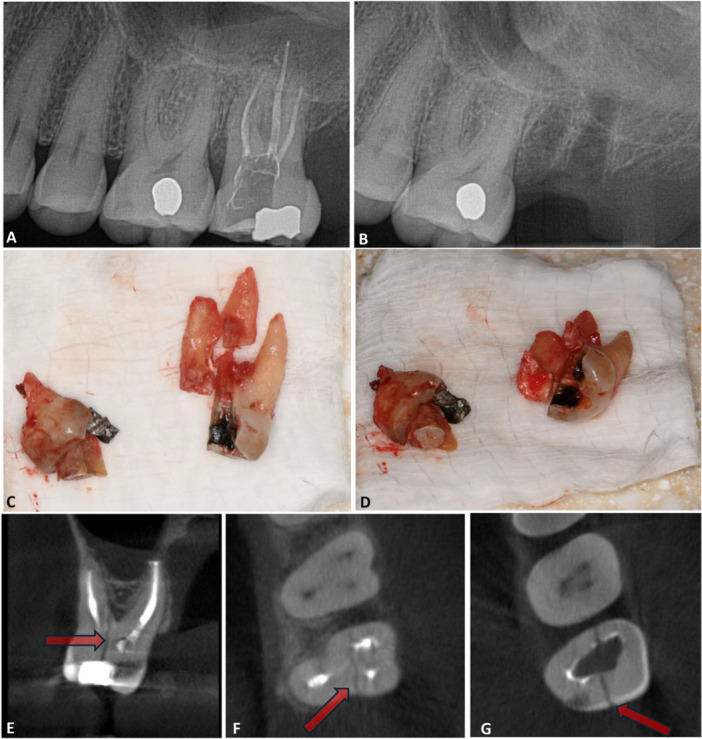
A case of a vertical root fracture in a patient's upper left second molar. Picture A: Pre‐operative periapical radiograph. Picture B: Post‐operative radiograph after extraction. Picture C: Fractured tooth in a similar orientation to the periapical radiograph. Picture D: Occluso‐apical view of the split tooth segments. Picture E: Sagittal view of the CBCT scan, the arrow indicates a VRF from the occlusal surface to the bifurcation area. Picture F: Axial view of the same CBCT at the level of the bifurcation area, the arrow indicates the area of the VRF where it terminates. Picture G: Axial view of the same CBCT at the level of the pulp chamber area, the arrow indicates the area of the VRF.

Management of cracks depending on the severity and extent could be using resin composite restorations by sealing cracks and/or incorporating extra‐coronal restorations (Pack 1994; Nicopoulou‐Karayianni et al. 1997; Cohen et al. 2006; Tamse et al. 1999; Sugaya et al. 2001; Meister et al. 1980; Habibzadeh et al. 2023; Lim et al. 2017; Lertchirakarn et al. 2003; Lustig et al. 2000; Mullally and Ahmed 2000; Patel et al. 2022; Corbella et al. 2014; Chang et al. 2016; PradeepKumar et al. 2021; Hu, Cao et al. 2022; Hu, Pan et al. 2022). However, the overall prognosis of a tooth with VRF is generally poor/hopeless, necessitating either extraction or removal of the fractured root (Pack 1994; Nicopoulou‐Karayianni et al. 1997; Cohen et al. 2006; Tamse et al. 1999; Meister et al. 1980; Habibzadeh et al. 2023; Lim et al. 2017; Lertchirakarn et al. 2003; Lustig et al. 2000; Mullally and Ahmed 2000; Patel et al. 2022; Corbella et al. 2014; Chang et al. 2016; PradeepKumar et al. 2021; Hu, Cao et al. 2022; Hu, Pan et al. 2022). Figure 1 represents a patient who was diagnosed with the VRF and treatment was carried out in the United States. This highlights the critical importance of early and accurate diagnosis. In this respect, the time interval between dental procedures and VRF detection was reported between 3.25 and 10 years (Pack 1994; Cohen et al. 2006; Chan et al. 1999; Meister et al. 1980; Hu, Pan et al. 2022).

Conventional radiographs show limited efficacy in diagnosing VRFs, especially in the early stages (Figure 1). This technique requires specific beam angulation and may fail to detect fractures if/when there is a fragment separation (Haupt et al. 2023; Torabinejad et al. 2020; Pack 1994; Rathke et al. 2022; Moule and Kahler 1999; Chai and Tamse 2012, 2018; von Arx et al. 2021; Walton 2017). Some radiographical characteristics include displacement of fractured fragments, radiolucent lines within the root canal system, periapical radiolucency in late stages mostly resembling J‐shaped patterns, and/or widening of the periodontal ligament space (Haupt et al. 2023; Torabinejad et al. 2020; Pack 1994; Rathke et al. 2022; Moule and Kahler 1999; Chai and Tamse 2012, 2018; von Arx et al. 2021; Walton 2017). However, the lack of accuracy in the signs and symptoms renders diagnosing VRFs challenging without advanced imaging techniques (Haupt et al. 2023; Mireku et al. 2010; Moule and Kahler 1999).

Cone beam computed tomography (CBCT) is a valuable tool for diagnosing VRFs, with more specificity in late stages, since this technique provides detailed three‐dimensional imaging of dental structures, facilitating comprehensive diagnostics (Figure 1) (Fuss et al. 2001). Despite its utility, there is insufficient evidence to suggest that CBCT is a reliable test for detecting VRFs, especially in teeth with intracanal materials (Khasnis et al. 2014; Rathke et al. 2022; Chai and Tamse 2012, 2018; von Arx et al. 2021; Walton 2017; Fuss et al. 2001; Haueisen et al. 2013). In addition, CBCT exposes patients to high radiation doses in comparison to conventional radiographs, raising concerns about its routine use (Fuss et al. 2001). A clinical presentation of both radiographs and CBCT can be seen in Figure 1.

Optical coherence tomography (OCT) is a non‐invasive imaging technique that has emerged as a potential technology for its accuracy and non‐invasive techniques (García‐Guerrero et al. 2018; Schweitzer et al. 1989). This system was developed in 1991 at MIT for Ophthalmology (García‐Guerrero et al. 2018). Subsequently, the successful in vivo measurements of the human retina in Vienna (1993), Zeiss‐Humphrey launched the first commercial OCT device in 1996, which allowed the introduction of this tool to dentistry (Huang et al. 1991; Fercher et al. 1993, 1995; Wilder‐Smith et al. 2008). The technique is based on low‐coherence interferometry, offering high‐resolution, cross‐sectional images of dental tissues that aimed to improve diagnostic accuracy and treatment planning (Machoy et al. 2017; Liao et al. 2021).

OCT uses low‐coherence interferometry utilizing near‐infrared light to capture detailed images of tissue microstructures. The light is split into two paths: one directed at the tissue and the other at a reference mirror (Vadivambal and Jayas 2015). The reflected light from both paths is combined to create an interference pattern, used to construct a high‐resolution cross‐sectional image (Sharma et al. 2015). Unlike ultrasound imaging, which uses sound waves, OCT employs light waves, resulting in superior resolution. The primary types of OCT are categorized based on their image acquisition methods:

- Time‐Domain OCT (TD‐OCT): The original OCT technique involves a moving reference mirror, that limits the speed and practicality of the device.
- Fourier‐Domain OCT (FD‐OCT): An advanced technique that eliminates the need for a moving reference mirror, allowing for faster and more efficient imaging. FD‐OCT includes:
  - Spectral‐Domain OCT (SD‐OCT): Uses a spectrometer to analyze the reflected light spectrum, enabling faster image acquisition (Vadivambal and Jayas 2015).
  - Swept‐Source OCT (SS‐OCT): Uses a tunable laser that sweeps through different wavelengths, providing a high signal‐to‐noise ratio and faster data acquisition (Vadivambal and Jayas 2015; Janjua et al. 2023).

OCT allows for the non‐invasive visualization of dental tissues such as enamel, dentin, and dental pulp, facilitating early detection of dental caries, assessment of tooth structure integrity, and monitoring of treatment progress (Machoy et al. 2017). Specific applications of OCT in clinical practice are listed in Table 1.

**Table 1:** Specific applications of OCT in dental practice.

Application fields	OCT use
Restorative dentistry/Cariology	Detection of early dental caries before radiographic appearance, monitoring caries progression post‐minimally invasive treatment, and assessment of enamel defects like Molar Incisor Hypoplasia and non‐carious tooth surface loss (Janjua et al. 2023).
Fixed prosthodontics	Evaluation of the fit and marginal integrity of fixed partial dentures, crowns, lumineers, and veneers to ensure proper fit and cementation (Sharma et al. 2015).
Implant dentistry	Assessment of implant proximity to vital structures, implant‐abutment fit, and peri‐implant tissue health, including detection of peri‐implant mucositis (Shemesh et al. 2007).
Periodontology	Visualization of periodontal structures, measurement of pockets and sulcus depths, and assessment of gingival regeneration post‐treatment (Liao et al. 2021).
Orthodontics	Evaluation of enamel surface characteristics post‐treatment and identification of adhesive remnants after debonding to prevent enamel damage (Janjua et al. 2023).
Oral pathology	Detection of dysplasia, vesiculobullous lesions, and malignancies. Offering real‐time, high‐resolution images for diagnosis and monitoring (Sharma et al. 2015).
Endodontics	Evaluation of internal tooth structures, such as cracks, fissures, and root fractures. Assessment of root canals during and after treatment (Shemesh et al. 2007).

**Figure 2:**
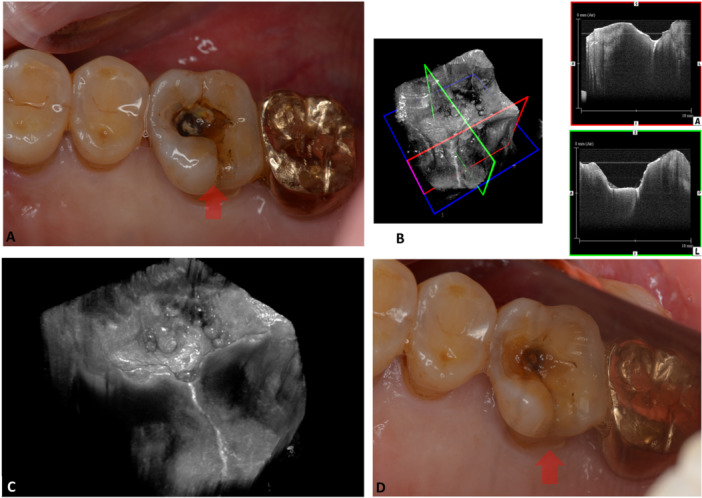
A scan of a crack in the upper right first molar using Swept Source Optical Coherence Tomography. Picture A: An intra‐oral pre‐operative photograph of the cracked tooth, with a distinct lesion at the occlusal area after the removal of Class 1 metal inlay (arrow). Picture B: 3D imaging of the first molar using SS‐OCT. 3D SS‐OCT system using high‐frequency swept laser light with a center wavelength of 1310 nm and a scanning range of 140 nm at 50 KHz (Yoshida Dental Mfg., Tokyo, Japan). Left: 3D image. Upper right: 2D image along the red square line in 3D image. Lower right: 2D image along the green square line in a 3D image. Dentin crack was imaged as a white line penetrating the cavity floor (arrows). Picture C: plain reconstruction without scan sections, with the location of the dentin crack clearly shown as a white line (arrows). Picture D: intraoral photograph of the same tooth after caries removal, where the presence of dentin crack was visibly confirmed (arrows).

OCT has been used previously in studies to diagnose dental cracks. Figure 2 presents a clinical use of OCT to detect cracks on clinical dental crowns. Despite its promise, OCT's current design is not yet optimized for widespread commercial use in endodontics to diagnose cases like VRFs (García‐Guerrero et al. 2018; Schweitzer et al. 1989). Challenges include limited penetration depth in dental hard tissues and the need for specialized equipment adaptations for intra‐oral use (Shemesh et al. 2007; Al Shehadat and Jain 2021; Imai et al. 2012; Schneider et al. 2017).

To be able to address this with future research, a thorough understanding of OCT's current innovations and limitations is required. Therefore, this scoping review aims to synthesize existing evidence on the application of OCT for detecting dental cracks and VRFs, with particular focus on its diagnostic performance, technological limitations, and potential for clinical adoption. The review also seeks to identify current gaps in the literature to inform future research.

## 2 Methodology

This review followed up the five stages of Scoping review by Mak and Thomas (2022):

1. Defining the research question,
2. Identifying relevant studies based on and criteria for eligibility,
3. Selecting studies,
4. Charting results,
5. Collecting, summarizing, and reporting data.

Subsequently, the PRISMA Extension for Scoping Reviews (PRISMA‐ScR) framework was employed (Tricco et al. 2018). The protocol was registered at the Open Science Framework and is available at the following link: https://osf.io/d8yhr.

### 2.1 Research Question

The research question was formulated based on the population, concept, and context framework (Pinto et al. 2023) with the eligibility criteria (PCC Framework):

**Population:** Teeth with cracks or VRFs

**Concept:** Use of OCT imaging

**Context:** In vitro, ex vivo, or clinical studies

**Study Design:** Original studies, theses, or book chapters published in the last 20 years

A scoping literature review was conducted to investigate the use of OCT as a diagnostic tool for cracks, including VRFs. The review aimed to address the question of “What do we currently know about OCT and what are the current gaps in the literature for it to be potentially used in diagnosing cracks, which may sometimes present as VRFs?”. To answer these questions broadly, specific terms in this review were used, including “Optical Coherence Tomography,” “Swept Source OCT,” and “Spectral Domain OCT,” with variations related to Endodontics, Periodontics, Endodontic‐Periodontics, and Cracks and VRFs (Tsesis et al. 2010).

### 2.2 Literature Search Strategy

A comprehensive search was conducted using PubMed, Scopus, Embase, EBSCOhost, and Google Scholar, covering publications up to January 2025 without any language restrictions. Table 2 represents the search strategies used to collect data from the various databases. Controlled vocabulary (e.g., MeSH, Emtree) and relevant free‐text keywords (e.g., “Optical Coherence Tomography,” “Swept Source OCT,” “Spectral Domain OCT,” “tooth cracks,” and “vertical root fractures”) were combined using Boolean operators.

**Table 2:** Search strategy for all databases used to collect data for this study.

Database	Search strategy
PubMed	(“Optical Coherence Tomography”[MeSH] OR “OCT” OR “Time‐Domain OCT” OR “TD‐OCT” OR “Fourier‐Domain OCT” OR “FD‐OCT” OR “Spectral‐Domain OCT” OR “SD‐OCT” OR “Swept‐Source OCT” OR “SS‐OCT”) AND (“Endodontics”[MeSH] OR “endodontics” OR “root canal” OR “VRF” OR “vertical root fracture”) AND (“Cracked teeth” OR “dental cracks” OR “tooth fractures” OR “enamel cracks” OR “root cracks”) OR (“Periodontics”[MeSH] OR “periodontics” OR “endodontic‐periodontics”).
Scopus	TITLE‐ABS‐KEY(“Optical Coherence Tomography” OR “OCT” OR “Time‐Domain OCT” OR “TD‐OCT” OR “Fourier‐Domain OCT” OR “FD‐OCT” OR “Spectral‐Domain OCT” OR “SD‐OCT” OR “Swept‐Source OCT” OR “SS‐OCT”) AND TITLE‐ABS‐KEY(“endodontics” OR “root canal” OR “VRF” OR “vertical root fracture”) AND TITLE‐ABS‐KEY(“cracked teeth” OR “dental cracks” OR “tooth fractures” OR “enamel cracks” OR “root cracks”) OR TITLE‐ABS‐KEY(“periodontics” OR “endodontic‐periodontics”).
EBSCOhost (CINAHL/Medline)	(TI OR AB)(“Optical Coherence Tomography” OR “OCT” OR “Time‐Domain OCT” OR “TD‐OCT” OR “Fourier‐Domain OCT” OR “FD‐OCT” OR “Spectral‐Domain OCT” OR “SD‐OCT” OR “Swept‐Source OCT” OR “SS‐OCT”) AND (TI OR AB)(“endodontics” OR “root canal” OR “VRF” OR “vertical root fracture”) AND (TI OR AB)(“cracked teeth” OR “dental cracks” OR “tooth fractures” OR “enamel cracks” OR “root cracks”) OR (TI OR AB)(“periodontics” OR “endodontic‐periodontics”).
Google Scholar	“Optical Coherence Tomography” OR “OCT” OR “Time‐Domain OCT” OR “TD‐OCT” OR “Fourier‐Domain OCT” OR “FD‐OCT” OR “Spectral‐Domain OCT” OR “SD‐OCT” OR “Swept‐Source OCT” OR “SS‐OCT” AND “endodontics” OR “root canal” OR “VRF” OR “vertical root fracture” AND “cracked teeth” OR “dental cracks” OR “tooth fractures” OR “enamel cracks” OR “root cracks” OR “periodontics” OR “endodontic‐periodontics”.
Embase (via Ovid)	(‘optical coherence tomography’/exp OR ‘optical coherence tomography’ OR ‘OCT’ OR ‘time‐domain oct’ OR ‘td‐oct’ OR ‘fourier‐domain oct’ OR ‘fd‐oct’ OR ‘spectral‐domain oct’ OR ‘sd‐oct’ OR ‘swept‐source oct’ OR ‘ss‐oct’) AND (‘endodontics’/exp OR ‘endodontics’ OR ‘root canal’ OR ‘VRF’ OR ‘vertical root fracture’) AND (‘cracked tooth’/exp OR ‘cracked teeth’ OR ‘dental cracks’ OR ‘tooth fractures’ OR ‘enamel cracks’ OR ‘root cracks’) OR (‘periodontics’/exp OR ‘periodontics’ OR ‘endodontic‐periodontics’).
Hand Search	Manually search contents of key endodontics, periodontics, dental imaging, and dental trauma journals and conference proceedings (e.g., Journal of Endodontics, International Endodontic Journal, Dental Traumatology) for articles mentioning: “Optical Coherence Tomography”/“OCT”/specific OCT types in relation to cracked teeth, root fractures, and endodontic‐periodontic lesions. Keywords to guide hand search: “OCT”, “root canal”, “VRF”, “tooth fracture”, “dental crack”, “periodontic lesion”.

In addition to peer‐reviewed articles, books and dissertations were included by searching ProQuest Dissertations and Theses Global, EBSCO Open Dissertations, and university repositories. Reference books were screened via dental specialty reading lists and library databases. Manual searches of specialty journals, conference abstracts, and reference lists of included articles ensured comprehensive coverage. Articles were included if they addressed OCT use in detecting dental cracks or VRFs and were published within the last 20 years (Tsesis et al. 2010; Haupt et al. 2023).

### 2.3 Study Screening

Three independent reviewers screened titles and abstracts, followed by full‐text assessment. Data related to the type of OCT, application field and method, authors and year of study, sample size, and key information, features, design, accuracy, and characteristics that were highlighted in each study were extracted from the studies being selected. Relevant articles were selected from databases and uploaded on EndNote software (Version 20.4, Clarivate) to keep track of references. Title and abstract reviews were carried out by all reviewers independently. Full text screening was conducted by the same reviewers and studies were assessed for eligibility for the review. If disagreements occurred, a fourth reviewer was identified to be approached so that consensus can be reached. All extracted articles were recorded in a Word document (DOCX, Version of Microsoft Word 2007 onward).

### 2.4 Data Extraction

Extracted data from all included studies assessed according to: authors’ names, year of study, study design, sample size, type of hard dental tissue, type of diagnostics and method of detection, outcomes assessed, author's conclusion(s), publication journals, and conflict of interest(s). Data were recorded in an Excel spreadsheet (XLSX, Version of Microsoft Excel 2007 onward).

Although this review was conducted as a scoping review, efforts were made to minimize potential sources of bias throughout the study selection and data retrieval process. To reduce selection bias, multiple electronic databases were systematically searched using broad search strategies, with no language restrictions applied. Three independent reviewers screened the titles and abstracts, followed by full‐text assessment, to ensure consistent and impartial inclusion decisions. The disagreements were resolved by discussing each study in details, and agreement was obtained. There was no need to approach the fourth reviewer. To address potential publication bias, unpublished studies such as theses, dissertations, and conference proceedings were considered, and reference lists of all included studies were hand‐searched for any additional relevant studies. In addition, the potential bias due to the inclusion of laboratory‐based studies was acknowledged as a limitation. While risk of bias assessment tools were not applied, the review methodology incorporated measures to enhance the transparency and reliability of the evidence synthesis. It should also be noted that a key difference between scoping and systematic reviews is the discussion of existing evidence regardless of methodological quality or risk of bias (Tricco et al. 2018). Therefore, this scoping review searched existing literature, identified gaps, and clarified concepts.

## 3 Results

### 3.1 Literature Search

According to the PRISMA flowchart (Figure 2):

- Records identified: 28,303
- After deduplication: 27,887
- Screened: 518
- Full‐text reviewed: 34
- Included: 10

All included studies were in vitro and published between 2008 and 2021. The systematic search identified 28,268 records from five databases: Google Scholar (*n* = 27,650), PubMed (*n* = 189), Embase (*n* = 224), Scopus (*n* = 120), and Ebsco (*n* = 85). A total of 35 records were identified through manual searches of reference lists. After the removal of 416 duplicate records and 27,369 irrelevant citations, 518 studies remained for screening.

Following the title and abstract screening, 482 studies were excluded for not meeting the inclusion criteria. Subsequently, a total of 36 full‐text studies were reviewed; however, two could not be accessed due to articles being dropped or unlocatable. Of the 34 full‐text studies assessed, 24 were excluded due to the following reasons: 5 were review articles, 9 focused on innovations without testing devices, and 10 were unrelated to endodontics.

A total of 10 studies met the inclusion criteria and were included in this review. These studies were published between 2008 and 2021 and focused on the use of OCT for detecting cracks in teeth, including VRF, enamel cracks, and apical root cracks. These were all laboratory‐based studies with sample sizes ranging from 6 to 80 teeth. Table 3 provides a detailed summary of the included studies, highlighting their objectives, methodologies, and key findings (Table 3).

**Table 3:** Current literature findings about OCT use for detecting cracks and VRF in the last 20 years.

Study	Study type	Sample size	Sensitivity	Specificity	Depth	Area of testing	Main findings
Shemesh et al. (2008)	In‐vitro	25 lower premolar teeth	0.93 overall, and a range between 0.91 and 0.95 for different observers.	0.95 overall, and a range between 0.89 and 1.00 for different observers	3.3 mm	VRF detection	OCT has the potential to detect VRF, especially with the use of intracanal catheter and after cleaning the canals. It stood out compared to visual and radiographical inspection, with a very good to excellent interobserver agreement, (kappa values of 0.7–0.9).
Imai et al. (2012)	In‐vitro	20 extracted human teeth	0.90 for whole‐thickness cracks and 0.95 for enamel surface cracks. All using SS‐OCT.	0.63 for whole‐thickness cracks and 0.75 for enamel surface cracks. All using SS‐OCT.	Was able to diagnose surface and whole‐thickness dentine cracks from the surface.	Enamel and whole‐thickness cracks	SS‐OCT offered superior detection to cracks compared to visual inspection. It provided detailed cross‐sectional images of enamel cracks, including their penetration depth. It could identify cracks extending beyond the dentinoenamel junction but with less accuracy. Interexaminer reproducibility was significantly higher for SS‐OCT (weighted kappa: 0.611) compared to visual inspection (weighted kappa: 0.178). While effective for coronal fractures, SS‐OCT has limitations in detecting root fractures in the subgingival zone due to probe design and signal attenuation in soft tissue and bone.
Nakajima et al. (2012)	In‐vitro	30 porcine premolars	N/S	N/S	Fractures up to 3 mm deep.	Incomplete crown fractures (cracks)	SS‐OCT was able to identify incomplete crown fractures and lamellae, and allowed their quantification. Cracks and lamellae disappear in SS‐OCT images when the line is parallel to the incidentlight and are brightest when they occur at 90° to the incident light.
Fried et al. (2014)	In‐vitro	N/S	N/S	N/S	3 mm teeth sections were used and were visibly clear.	Enamel cracks	OCT was able to identify superficial cracks’ presence but its utility was limited for assessing deeper cracks. Near‐InfraRed transillumination at 1300 nm was found to leverage the high transparency of dental enamel for clear visualization of cracks through disruption of light propagation through enamel. Integrating near‐IR imaging with other diagnostic technologies like OCT or ultrasound could offer the possibility to address their limitations.
Lee et al. (2016)	In‐vitro	61 teeth from 12 cadavers, 12 incisors, 23 premolars, and 26 molars without extensive restoration and dental caries	N/S	N/S	About 3 mm.	Detection of cracked‐tooth syndrome	SS‐OCT detected cracks in 65.1% of examined surfaces, surpassing visual inspection (47.4%) and trans‐illumination (52.3%). It had the highest mean number of detected crack lines per surface at 86.29%, outperforming trans‐illumination (78.80%), visual inspection (48.86%), and micro‐CT (60.11%). Along its ability to distinguish structural cracks from craze lines, it could identify cracks that were undetectable by trans‐illumination or micro‐CT in some cases.
de Oliveira et al. (2017)	In‐vitro	20 extracted human single‐rooted mandibular incisors with no open apices, previous endodontic treatment, dental calculus, hypercementosis, internal/external resorption, or root caries	0.83 for SS‐OCT and 0.917 for SD‐OCT	0.75 for SS‐OCT and 0.875 for SD‐OCT	Approximately 3 mm.	Detection of apical root cracks	SD‐OCT and SS‐OCT successfully detected apical dentinal microcracks. Although SD‐OCT showed better detection than SS‐OCT, this was not statistically significant. Interexaminer agreement ranged from substantial to almost perfect for both systems, with SD‐OCT showing slightly better consistency. Both systems had limitation with the penetration depth.
Kim et al. (2017)	In‐vitro	6 teeth of various canal numbers were subjected to stress similar to natural masticatory stresses in‐vitro and then evaluated	N/S	N/S	Only surface cracks, not deep cracks beyond 2–3 mm	Detection of craze lines, crack lines, split tooth, and VRF.	SS‐OCT provides superior resolution (approximately 10 μm/pixel) in comparison with CBCT or intraoral X‐ray imaging, without a risk of radioactivity exposure. It was able to detect easily cracks and craze lines, compared to the radiographic modalities and transillumination, and the software automatic detection tool proposed in this study further eases the detection. However, the lack of depth of OCT limited the ability to identify whether the tooth is split or if the crack extends deep to be defined as VRF.
Segarra et al. (2017)	In‐vitro	80 teeth, 10 of incisors, cuspids, bicuspids, and molars from each jaw	N/S	N/S	Cracks originating from surface enamel. No depth specified.	Enamel crack behavior	SS‐OCT had the ability to detect cracks and assist in determining its pattern in whole human teeth. OCT offers potential applications in early diagnosis, monitoring, and guiding the treatment of tooth cracks in clinical settings.
Shimada et al. (2020)	In‐vitro	20 extracted human teeth	0.98 for enamel demineralization and 0.60 for dentin caries, both using SS‐OCT	0.75 for enamel demin, compared remineralization and 0.98 for dentin caries, both using SS‐OCT	Limited to the coronal portion where the laser light can be irradiated.	Evaluation of dental caries, tooth crack, and age‐related changes in tooth structure	SS‐OCT was able to detect cracks and separate layers unlike transillumination that may not identify crack depth, and other current diagnostic methods that fail to detect tooth cracks, especially in early stages. Inter‐examiner reproducibility (measured by weighted kappa‐values) was significantly higher for SS‐OCT (0.61) than for transillumination (0.18). However, the current design limits SS‐OCT to primarily to the coronal portion of the tooth due to the penetration depth of IR‐light.
Birsch (2021)	In‐vitro	33 sterilized teeth, 11 with single rooted and 12 with multi‐rooted teeth	N/S	N/S	Circumferential of the inner root surface. Depth was not specified.	Prepared root canal space, resorptive defects, small perforation sites, and fracture lines.	OCT introduced by catheter into the root canal system offers an unparalleled imaging modality for detecting intact dentin walls, variations of lumen‐wall integrity, man‐made defects, cracks, perforations, and resorptive‐like areas of interest. Although OCT requires certain width and canal preparation, it remains superior to US which was not feasible to introduce into a root canal system.

### 3.2 Study Characteristics

**Figure 3:**
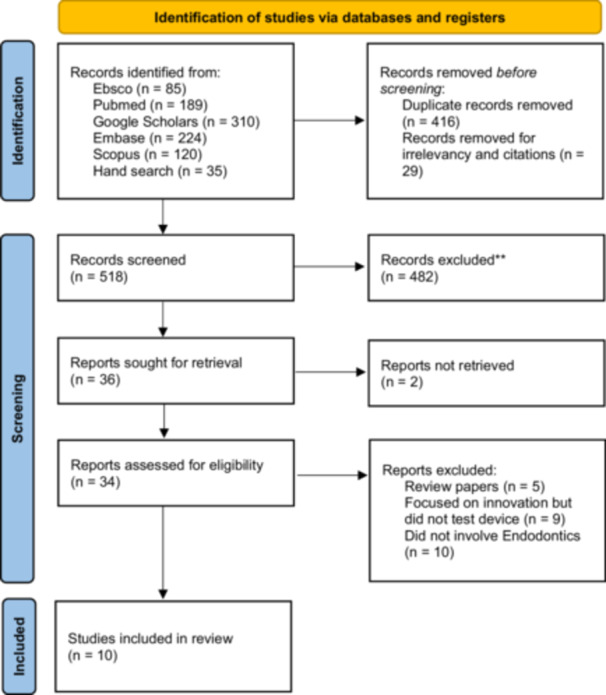
Flow diagram of the literature search and included studies.

The use of OCT has become an additional option in clinical endodontics for diagnosing conditions such as cracks and fractures in the last 20 years (Imai et al. 2012; Shemesh et al. 2008; Nakajima et al. 2012; Segarra et al. 2017; de Oliveira et al. 2017; Shimada et al. 2020; Birsch 2021; Fried et al. 2014; Kim et al. 2017; Lee et al. 2016). Table 3 represents key findings of the included studies. Two types of OCT delivery were introduced in this review; the first group discussed the use of OCT during root canal treatment by introducing the tool into the root canal system (Shemesh et al. 2008; Birsch 2021). The rest of the studies focused on the OCT to explore cracks on the tooth surface instead of inserting probes into the root canal system (Imai et al. 2012; Nakajima et al. 2012; Segarra et al. 2017; de Oliveira et al. 2017; Shimada et al. 2020; Fried et al. 2014; Kim et al. 2017; Lee et al. 2016). This could be a non‐invasive method as performed by Imai et al. (2012), Nakajima et al. (2012), Lee et al. (2016), Kim et al. (2017), Segarra et al. (2017), and Shimada et al. (2020) to assess cracks on clinical crowns (Figure 3) (Imai et al. 2012; Nakajima et al. 2012; Segarra et al. 2017; Shimada et al. 2020; Kim et al. 2017; Lee et al. 2016). Alternatively, this could be invasive as performed by Fried et al. (2012) in their study where they prepared 3 mm sections to the tooth. Another study by de Oliveira et al. (2017) accessed the pulp chamber and prepared the root canal systems to 10 ISO K hand‐file, and carried out root canal therapy (de Oliveira et al. 2017; Fried et al. 2014). Overall, studies that reported OCT's sensitivity and specificity often showed high sensitivity (83%–98%) and medium to high specificity (63%–100%) in detecting cracks and VRFs (Table 3).

## 4 Discussion

### 4.1 Key Findings

This scoping review aimed to provide a comprehensive synthesis of all available evidence on OCT in terms of cracks and VRF detection in order to offer clinicians and researchers evidence‐based approach and decision‐making process regarding the adoption and further development of OCT technology in endodontics (Rivera and Walton 2008; Tsesis et al. 2010; Sugaya et al. 2001; Meister et al. 1980; Habibzadeh et al. 2023). Sensitivity and specificity were the metrics used in some of the included studies to evaluate the diagnostic performance of OCT when compared to other tools such as clinical assessments, radiographs, CBCT (Table 3). Sensitivity refers to the ability of a diagnostic tool to correctly identify true positives. In this case, detecting the presence of cracks or VRFs when they are truly present. Specificity measures the ability to correctly identify true negatives, or cases where no cracks or fractures exist. High sensitivity reduces the likelihood of missed diagnoses, while high specificity minimizes false positives. The superior sensitivity of FD‐OCT over the TD‐OCT, marks a transition in use (Chopra et al. 2021; Bezerra et al. 2009). Shemesh et al. (2008) were the first to develop a model and test for cracks, specifically for VRF, using a rotating optical fiber probe, the “M2‐CV OCT system with an ImageWire 2 catheter,” inside the root canal system. This technique utilized the TD‐OCT by providing detailed imaging with a high overall specificity (95%) and sensitivity (93%) in detecting VRFs (Table 3). The preference for the FD‐OCT also witnessed a shift from the SD‐OCT towards the SS‐OCT since the new system offers high imaging speeds and long wavelengths, enabling deeper penetration into dental tissues (sometimes exceeding 3 mm when compared to 1–3 mm with the use of SD‐OCT), and optimum tissue contrast (Imai et al. 2012; Schneider et al. 2017; Nakajima et al. 2012; de Oliveira et al. 2017; Adegun et al. 2013). However, the challenge related to the penetration depth remains unresolved. Current dental OCT systems typically operate at near‐infrared wavelengths below 1000 nm, offering approximately 1–3 mm of penetration in hard tissues (Imai et al. 2012; Schneider et al. 2017; Nakajima et al. 2012; de Oliveira et al. 2017; Adegun et al. 2013). Near‐InfraRed transillumination at 1300 and 1310 nm was found to leverage the high transparency of dental enamel for clear visualization of cracks through disruption of light propagation through enamel (Fried et al. 2014; Kim et al. 2017). While adequate for surface and coronal imaging, this penetration depth is insufficient for endodontic applications particularly when the OCT probe cannot be positioned inside the root canal system to image internal structures. Imai et al. (2012) reported a sensitivity of 0.90 for deep enamel cracks in comparison to 0.95 for superficial ones, and specificity values around 0.63 and 0.75, respectively (Imai et al. 2012). These values confirm that the thicker the enamel and dentine gets, the lower values of sensitivity and specificity are. This was later supported by Shimada et al. (2020) (Table 2) (Shimada et al. 2020). The only study to oppose this finding was de Oliveira et al. (2017), which reported high analytical performance in SD‐OCT over SS‐OCT (sensitivity of 0.917:0.833, specificity of 0.875:0.750 and accuracy of 0.9:0.8 for SD‐OCT and SS‐OCT respectively), however, the results were statistically insignificant (de Oliveira et al. 2017).

### 4.2 Limitations of Evidence

OCT's non‐invasive nature and ability to provide high‐resolution, real‐time imaging without radiation exposure provides a valued asset in endodontic diagnostics (Machoy et al. 2017; Sharma et al. 2015; Janjua et al. 2023). To achieve that, a suitable design needs to be introduced as an initial step in adopting this system into clinical endodontics. Current OCT systems have a penetration depth typically reaching only 1–3 mm, which may be insufficient for visualizing deeper structures within the root (Imai et al. 2012; Schneider et al. 2017). Currently, catheter‐based OCTs, such as the M2‐CV and intravascular probes in Shemesh et al. (2008) or the commercially available catheter‐based probes used in Birsch's (2021) study, have been introduced for root canal imaging, however, these systems required canal preparation to a K file of ISO size 50–90, as the imaging probe is too big to be accommodated in unprepared root canal systems (Shemesh et al. 2008; Birsch 2021).

Alternative tools were designed for root canal system however targeted only to the surface of the tooth (Shemesh et al. 2007; Imai et al. 2012; Shemesh et al. 2008; Nakajima et al. 2012; Birsch 2021). Based on the findings of this review, further research related to the system design are required to visualize root canal systems as that seems to hold the key to addressing the penetration‐depth limitation of OCT. In addition, the existing devices require further miniaturization and ergonomic design to be practical for routine clinical use in endodontics (Shemesh et al. 2007; Al Shehadat and Jain 2021). Few solutions were suggested to address this issue, such as making OCT in the form of handheld mobile OCT devices and integrations with dental intra‐oral scanners (Imai et al. 2012; Shimada et al. 2020; Chopra et al. 2021; Iino et al. 2014). In addition, exploring the potential of artificial intelligence in interpreting OCT images could further enhance the diagnostic accuracy and efficiency as seen in OCT's design by Hu et al. (2022a) and Kim et al. (2017).

### 4.3 Comparing OCT With Different Imaging Modalities

Longitudinal studies would be crucial to understand the long‐term benefits of OCT‐guided diagnosis and treatment in endodontics, including its impact on prognostic outcomes (Cohen et al. 2006; Patel et al. 2022). All reported papers in this scoping review have been conducted in vitro, highlighting the need for controlled randomized clinical trials to validate its efficacy and practicality in endodontic settings (Shemesh et al. 2007, 2008; Imai et al. 2012; Nakajima et al. 2012; Segarra et al. 2017; de Oliveira et al. 2017; Birsch 2021; Fried et al. 2014; Kim et al. 2017; Lee et al. 2016). Further research could also compare OCT with other advanced imaging modalities, which are necessary to establish the relative diagnostic accuracy, clinical outcomes, and cost‐effectiveness (Al Shehadat and Jain 2021; Suassuna et al. 2018; Ţogoe et al. 2021). The current available landscape of dental imaging technologies presents various options, ranging from ultrasound to radiographs, CBCT, Micro‐CT, and OCT, each with its unique strengths and limitations. Table 4 presents a comparison between the previously mentioned tools according to literature and the current OCT systems utilized in the scope of this paper (Table 4) (Al Shehadat and Jain 2021; Birsch 2021; Suassuna et al. 2018; Ţogoe et al. 2021). In addition, Figures 1 and 2 represent a clinical comparison between OCT and radiographs and CBCT for two cases with dental cracks.

**Table 4:** Comparison of various endodontic diagnostic tools.

Technology	Strengths	Limitations	Applications in endodontics
Ultrasound	Non‐radiative.	Limited efficacy in visualizing hard tissues, therefore, this system is less useful for that area in comparison to radiographs, CBCT, and OCT.	Real‐time assessment of soft tissue conditions.
	Beneficial for soft tissue imaging.	In endodontics, it needs excessive canal preparation compared to the catheterized OCT prob (ISO size 130 file for US vs. size 90 for OCT). Therefore, it requires development of smaller, more flexible probes for root canal navigation.	Evaluation of periapical tissues and inflammatory conditions.
Radiographs	Accessible and cost‐effective.	Generates static, 2D images lacking depth information when compared to CBCT, MicroCT, and OCT.	Widely used for initial diagnostics and assessing periapical conditions.
	Available in conventional and digital formats. Moreover, digital format offers image enhancements.	Limited in identifying certain observations (i.e., voids in root canal fillings) when compared to CBCT, MicroCT, and OCT.	Integral to dental diagnostics despite limitations.
		Utilizes radiation.	Potential optimization when integrated with OCT for better resolution and contrast.
CBCT (cone‐beam computed tomography) and MicroCT (micro‐computed tomography).	Detailed 3D imaging of dental structures.	Higher radiation exposure, requiring adherence to the ALARA principle to minimize radiation exposure.	Essential for complex diagnostic cases, guided procedures, and surgeries.
	High‐resolution images with multiplane imaging and dynamic data correction capabilities.	More expensive than traditional radiographs. Therefore, the system is unable to substitute the radiographs during initial screening.	Indispensable for visualizing calcified canals, hard tissues, and minute structural details.
	Exceptional spatial resolution (~ 10–15 µm).		MicroCT is used in vivo and in vitro for assessing root canal obturation and detecting anomalies like voids.
OCT (optical coherence tomography)	High‐resolution imaging.	Limited in visualizing soft tissue structures compared to Micro‐CT.	Promising tool for real‐time imaging and extending beyond in vitro studies.
	Real‐time capabilities.	Currently outperformed by micro‐CT in soft tissue visualization.	Potentially beneficial for optimizing dental X‐ray parameters when combined with radiographs.
	Portability in recent designs such hand‐held machines.	Requires canal preparation to somewhere between ISO 50 and 90 K files.	Future potential in endodontics for detailed dental pathologies and treatment outcomes.
	In‐vitro studies reported sensitivity and specificity for detecting cracks and VRF between (63%–100%) and (83%–98%), respectively. Both of which are higher than any other tool.		

Within the limitations of this review, a total of 10 papers were found to be relevant to the OCT use in cracked teeth and VRFs. Besides cracks and VRFs, OCT has been utilized to visualize multiple areas of surface discontinuity in the tooth, including cavities from dental caries in enamel and dentin layers, endodontic anatomy and walls, additional canals like MB2, gaps between root canal fillings and canal walls or root apex (Shemesh et al. 2007, 2008 Imai et al. 2012; Nakajima et al. 2012; Segarra et al. 2017; de Oliveira et al. 2017; Shimada et al. 2020; Birsch 2021; Iino et al. 2014; Suassuna et al. 2018; Ţogoe et al. 2021; Braz et al. 2009; Li et al. 2021; Minamino et al. 2015). Until these findings have been addressed, OCT is unable to substitute other technologies like CBCT (the current gold standard) for clinical use such as diagnosing VRFs as there is 20 years’ worth of research and utilization between the first introduction of both technologies into clinical practice (Wilder‐Smith et al. 2008; Bromberg and Brizuela 2023). Despite these challenges, the use of OCT in endodontics could be promising. Ongoing technological advancements are actively addressing these limitations. Researchers are currently developing new OCT systems that incorporate long wavelengths (> 1300 nm) to increase penetration depth (Janjua et al. 2023). The emergence of handheld and mobile OCT devices, as well as catheter‐based systems for intracanal use, is making OCT accessible for endodontic applications (Shemesh et al. 2007, 2008; Birsch 2021; Chopra et al. 2021). Furthermore, efforts to integrate OCT with other imaging modalities or incorporate it into existing dental equipment, such as intraoral scanners, may significantly enhance its utility in clinical practice (Kim et al. 2024). The issue of bulky and expensive units is nearing resolution (Janjua et al. 2023; Chopra et al. 2021; Kim et al. 2024). With clinical evidence, OCT may become a routine tool in endodontic practice and standard detection aid for orofacial conditions and lesions.

### 4.4 Clinical Implications

This scoping review confirms the diagnostic potential of OCT for detecting dental cracks and VRFs while exposing key gaps in the existing literature. It should be noted that this review recognizes a limitation with regards to the included studies which were conducted in vitro, since this might potentially introduce a performance bias as the diagnostic purpose of OCT in controlled laboratory conditions might not fully reflect its effectiveness or challenges in actual clinical settings. Therefore, the current evidence is limited to laboratory‐based studies with variable methodologies and limited clinical trials, restricting its immediate clinical application. In addition, inconsistencies in the OCT system types, imaging protocols, and outcome measures hinder direct comparison across the studies.

The OCT technology is promising. In particular, catheter‐based and handheld OCT designs could be adapted for future intraoral applications. However, the logistical barriers need to be considered for further research. First, a high‐quality OCT system with a good penetration depth is relatively expensive, limiting their accessibility to many dental practices (Janjua et al. 2023). Second, although OCT systems have been introduced to clinical practice, the interpretation of OCT scans requires specialized training, which is not yet standardized in the dental curriculum. This could be largely due to the lack of a feasible design to use in the clinical dental setup. Third, the existing OCT devices are too bulky or rigid for effective use in posterior teeth or root canal systems. It should be noted that the current OCT machines are only designed for surface scans like enamel and clinical crowns. Finally, the intra‐oral use of this system may be restricted by probe size, patient tolerance, and accessibility to deeper regions of the mouth. In clinical practice, field sizes of several centimeters are often required, and clinicians may require taking multiple images to completely capture a lesion of that size. In this respect, this could be cumbersome, time‐consuming, and uncomfortable for both the clinician and patient (Janjua et al. 2023).

Further research could prioritize well‐designed controlled randomized clinical trials, standard operating procedures, and development of clinically adaptable OCT devices. Addressing these gaps is essential to advancing OCT from experimental use toward evidence‐based integration in endodontic diagnostics.

The prevalence of VRFs in extracted endodontically treated teeth ranges from 2% to 20%, highlighting significant clinical variability (Lee et al. 2023; Rathke et al. 2022; Mireku et al. 2010). The prognosis of a tooth with VRF is generally poor/hopeless at late stages, necessitating either extraction or removal of the fractured root (Pack 1994; Nicopoulou‐Karayianni et al. 1997; Cohen et al. 2006; Tamse et al. 1999; Meister et al. 1980; Habibzadeh et al. 2023; Lim et al. 2017; Lertchirakarn et al. 2003; Lustig et al. 2000; Mullally and Ahmed 2000; Patel et al. 2022; Corbella et al. 2014; Chang et al. 2016; PradeepKumar et al. 2021; Hu et al. 2022a, 2022b). A well‐designed OCT that is optimized for clinical and endodontic practice may give a solution to VRF by establishing early diagnosis and thus allowing early intervention before the crack expands to a late non‐restorable stage, which may allow certain treatments that show evidence of success at early stages incorporation of resin composite and/or extra‐coronal restorations (Sugaya et al. 2001).

## 5 Conclusions

Within the limitation of this scoping review, the use of OCT is promising for the detection of cracks and VRFs. By addressing the limitations related to penetration depth, mechanical design, and soft tissue imaging, OCT may find its path into clinical adoption, including clinical endodontics. Areas that can help in this include developing OCT systems tailored for routine endodontic use and assessing the long‐term impacts in the field. OCT holds a promising sustainable solution for an accurate, efficient, and user/patient‐friendly diagnostic procedure, marking a significant step forward in the field of clinical endodontics.

## Author Contributions

**MHD. Mouaffak Alkhani** and **Ahmad Yaser Albittar:** literature search, references collection. **MHD. Mouaffak Alkhani**, **Ahmad Yaser Albittar**, and **Uzma Munawwar Shaikh:** reviewing and summarizing searched literature. **MHD. Mouaffak Alkhani:** original draft preparation. **MHD. Mouaffak Alkhani**, **Ahmad Yaser Albittar**, **Muhammad Takriti**, and **Aylin Baysan:** conceptualization. **Muhammad Takriti** and **Aylin Baysan:** supervision of the project, analysis and interpretation, reviewing and editing.

## Funding

The authors received no specific funding for this work.

## Conflicts of Interest

The authors declare no conflicts of interest.

## Supporting information

Supplementary Information

## Data Availability

All information presented in this article can be found in the following databases; PubMed, Scopus, Ebsco, Google Scholars, and Embase. Data sharing is not applicable to this article, as no new data were created or analyzed.

## References

[cre270323-bib-0001] Adegun, O. K. , P. H. Tomlins , E. Hagi‐Pavli , D. L. Bader , and F. Fortune . 2013. “Quantitative Optical Coherence Tomography of Fluid‐Filled Oral Mucosal Lesions.” Lasers in Medical Science 28: 1249–1255. 22996049 10.1007/s10103-012-1208-y

[cre270323-bib-0002] Al Shehadat, S. , and P. Jain . 2021. “Digitalization in Endodontics.” In Digitization in Dentistry: Clinical Applications, 89–139. Springer International Publishing.

[cre270323-bib-0003] von Arx, T. , P. Maldonado , and M. M. Bornstein . 2021. “Occurrence of Vertical Root Fractures After Apical Surgery: A Retrospective Analysis.” Journal of Endodontics 47, no. 2: 239–246. 33098890 10.1016/j.joen.2020.10.012

[cre270323-bib-0004] Bezerra, H. G. , M. A. Costa , G. Guagliumi , A. M. Rollins , and D. I. Simon . 2009. “Intracoronary Optical Coherence Tomography: A Comprehensive Review Clinical and Research Applications.” JACC Cardiovascular interventions 2, no. 11: 1035–1046. 19926041 10.1016/j.jcin.2009.06.019 PMC4113036

[cre270323-bib-0005] Birsch, R. E. 2021. *In Vitro Evaluation of Optical Coherence Tomography and Ultrasound Probes Used in the Detection of Intact Dentin and Various Anomalies Within Root Canals* . Boston University.

[cre270323-bib-0006] Braz, A. K. S. , B. B. C. Kyotoku , and A. S. L. Gomes . 2009. “In Vitro Tomographic Image of Human Pulp‐Dentin Complex: Optical Coherence Tomography and Histology.” Journal of Endodontics 35, no. 9: 1218–1221. 19720219 10.1016/j.joen.2009.05.003

[cre270323-bib-0007] Bromberg, N. , and M. Brizuela. Dental Cone Beam Computed Tomography. 2023. 37276309

[cre270323-bib-0008] Chai, H. , and A. Tamse . 2012. “Fracture Mechanics Analysis of Vertical Root Fracture From Condensation of Gutta‐Percha.” Journal of Biomechanics 45, no. 9: 1673–1678. 22503579 10.1016/j.jbiomech.2012.03.022

[cre270323-bib-0009] Chai, H. , and A. Tamse . 2018. “Vertical Root Fracture in Buccal Roots of Bifurcated Maxillary Premolars From Condensation of Gutta‐Percha.” Journal of Endodontics 44, no. 7: 1159–1163. 29861061 10.1016/j.joen.2018.03.017

[cre270323-bib-0010] Chan, C.‐P. , C.‐P. Lin , S.‐C. Tseng , and J.‐H. Jeng . 1999. “Vertical Root Fracture in Endodontically Versus Nonendodontically Treated Teeth: A Survey of 315 Cases in Chinese Patients.” Oral Surgery, Oral Medicine, Oral Pathology, Oral Radiology, and Endodontology 87, no. 4: 504–507. 10.1016/s1079-2104(99)70252-0 10225635

[cre270323-bib-0011] Chang, E. , E. Lam , P. Shah , and A. Azarpazhooh . 2016. “Cone‐Beam Computed Tomography for Detecting Vertical Root Fractures in Endodontically Treated Teeth: A Systematic Review.” Journal of Endodontics 42, no. 2: 177–185. 26631300 10.1016/j.joen.2015.10.005

[cre270323-bib-0012] Chopra, R. , S. K. Wagner , and P. A. Keane . 2021. “Optical Coherence Tomography in the 2020s—Outside the Eye Clinic.” Eye 35, no. 1: 236–243. 33168975 10.1038/s41433-020-01263-6 PMC7853067

[cre270323-bib-0013] Cohen, S. , L. Berman , L. Blanco , L. Bakland , and J. Kim . 2006. “A Demographic Analysis of Vertical Root Fractures.” Journal of Endodontics 32, no. 12: 1160–1163. 17174672 10.1016/j.joen.2006.07.008

[cre270323-bib-0014] Corbella, S. , M. Del Fabbro , A. Tamse , E. Rosen , I. Tsesis , and S. Taschieri . 2014. “Cone Beam Computed Tomography for the Diagnosis of Vertical Root Fractures: A Systematic Review of the Literature and Meta‐Analysis.” Oral Surgery, Oral Medicine, Oral Pathology and Oral Radiology 118, no. 5: 593–602. 25442497 10.1016/j.oooo.2014.07.014

[cre270323-bib-0015] Ellis, S. G. S. 2001. “Incomplete Tooth Fracture–Proposal for a New Definition.” British Dental Journal 190, no. 8: 424–428. 11352390 10.1038/sj.bdj.4800992

[cre270323-bib-0016] Fercher, A. F. , C. K. Hitzenberger , W. Drexler , G. Kamp , and H. Sattmann . 1993. “In Vivo Optical Coherence Tomography.” American Journal of Ophthalmology 116: 113–114. 8328536 10.1016/s0002-9394(14)71762-3

[cre270323-bib-0017] Fercher, A. F. , C. K. Hitzenberger , G. Kamp , and S. Y. El‐Zaiat . 1995. “Measurement of Intraocular Distances by Backscattering Spectral Interferometry.” Optics Communications 117, no. 1–2: 43–48.

[cre270323-bib-0018] Fried, W. A. , J. C. Simon , S. Lucas , et al. 2014. Editors. “Near‐IR imaging of cracks in teeth.” In *Proceedings of SPIE‐‐the International Society for Optical Engineering*. 10.1117/12.2045686 PMC4013098 24817806

[cre270323-bib-0019] Fuss, Z. , J. Lustig , A. Katz , and A. Tamse . 2001. “An Evaluation of Endodontically Treated Vertical Root Fractured Teeth: Impact of Operative Procedures.” Journal of Endodontics 27, no. 1: 46–48. 11487164 10.1097/00004770-200101000-00017

[cre270323-bib-0020] García‐Guerrero, C. , C. Parra‐Junco , S. Quijano‐Guauque , N. Molano , G. A. Pineda , and D. J. Marín‐Zuluaga . 2018. “Vertical Root Fractures in Endodontically‐Treated Teeth: A Retrospective Analysis of Possible Risk Factors.” Journal of Investigative and Clinical Dentistry 9, no. 1: e12273. 10.1111/jicd.12273 28474492

[cre270323-bib-0021] Habibzadeh, S. , Z. Ghoncheh , P. Kabiri , and S. A. Mosaddad . 2023. “Diagnostic Efficacy of Cone‐Beam Computed Tomography for Detection of Vertical Root Fractures in Endodontically Treated Teeth: A Systematic Review.” BMC Medical Imaging 23, no. 1: 68. 37264339 10.1186/s12880-023-01024-3 PMC10236739

[cre270323-bib-0022] Haueisen, H. , K. Gärtner , L. Kaiser , D. Trohorsch , and D. Heidemann . 2013. “Vertical Root Fracture: Prevalence, Etiology, and Diagnosis.” Quintessence International 44, no. 7: 467–474. 23757466 10.3290/j.qi.a29715

[cre270323-bib-0023] Haupt, F. , A. Wiegand , and P. Kanzow . 2023. “Risk Factors for and Clinical Presentations Indicative of Vertical Root Fracture in Endodontically Treated Teeth–A Systematic Review and Meta‐Analysis.” Journal of Endodontics 49: 940–952. 37307871 10.1016/j.joen.2023.06.004

[cre270323-bib-0024] Hu, Z. , D. Cao , Y. Hu , et al. 2022. “Diagnosis of In Vivo Vertical Root Fracture Using Deep Learning on Cone‐Beam CT Images.” BMC Oral Health 22, no. 1: 382. 36064682 10.1186/s12903-022-02422-9 PMC9446797

[cre270323-bib-0025] Hu, Z. , X. Pan , Y. Hu , et al. 2022. “Exploring the Use of Enhanced Cone‐Beam CT Technique to Diagnose Vertical Root Fracture.” Journal of the Mechanical Behavior of Biomedical Materials 130: 105175. 35320764 10.1016/j.jmbbm.2022.105175

[cre270323-bib-0026] Huang, D. , E. A. Swanson , C. P. Lin , et al. 1991. “Optical Coherence Tomography.” Science 254, no. 5035: 1178–1181. 1957169 10.1126/science.1957169 PMC4638169

[cre270323-bib-0027] Iino, Y. , A. Ebihara , T. Yoshioka , et al. 2014. “Detection of a Second Mesiobuccal Canal in Maxillary Molars by Swept‐Source Optical Coherence Tomography.” Journal of Endodontics 40, no. 11: 1865–1868. 25266471 10.1016/j.joen.2014.07.012

[cre270323-bib-0028] Imai, K. , Y. Shimada , A. Sadr , Y. Sumi , and J. Tagami . 2012. “Noninvasive Cross‐Sectional Visualization of Enamel Cracks by Optical Coherence Tomography In Vitro.” Journal of Endodontics 38, no. 9: 1269–1274. 22892749 10.1016/j.joen.2012.05.008

[cre270323-bib-0029] Janjua, O. S. , W. Jeelani , M. I. Khan , et al. 2023. “Use of Optical Coherence Tomography in Dentistry.” International Journal of Dentistry 2023, no. 1: 4179210. 38111754 10.1155/2023/4179210 PMC10727803

[cre270323-bib-0030] Khasnis, S. , K. Kidiyoor , A. Patil , and S. Kenganal . 2014. “Vertical Root Fractures and Their Management.” Journal of Conservative Dentistry 17, no. 2: 103–110. 24778502 10.4103/0972-0707.128034 PMC4001262

[cre270323-bib-0031] Kim, H. , H. Cho , W. Lee , et al. 2024. “Development of Handheld Optical Coherence Tomography Based on Commercial Intra‐Oral Scanner Shape for Extended Clinical Utility in Dentistry.” International Journal of Imaging Systems and Technology 34, no. 1: e23024.

[cre270323-bib-0032] Kim, J.‐M. , S.‐R. Kang , and W.‐J. Yi . 2017. “Automatic Detection of Tooth Cracks in Optical Coherence Tomography Images.” Journal of Periodontal & Implant Science 47, no. 1: 41–50. 28261523 10.5051/jpis.2017.47.1.41 PMC5332334

[cre270323-bib-0033] Lee, K. , M. Ahlowalia , R. P. Alfayate , S. Patel , and F. Foschi . 2023. “Prevalence of and Factors Associated With Vertical Root Fracture in a Japanese Population: An Observational Study on Teeth With Isolated Periodontal Probing Depth.” Journal of Endodontics 49, no. 12: 1617–1624. 37660764 10.1016/j.joen.2023.08.018

[cre270323-bib-0034] Lee, S.‐H. , J.‐J. Lee , H.‐J. Chung , J.‐T. Park , and H.‐J. Kim . 2016. “Dental Optical Coherence Tomography: New Potential Diagnostic System for Cracked‐Tooth Syndrome.” Surgical and Radiologic Anatomy 38: 49–54. 26168856 10.1007/s00276-015-1514-8

[cre270323-bib-0035] Lertchirakarn, V. , J. Palamara , and H. Messer . 2003. “Patterns of Vertical Root Fracture: Factors Affecting Stress Distribution in the Root Canal.” Journal of Endodontics 29, no. 8: 523–528. 12929700 10.1097/00004770-200308000-00008

[cre270323-bib-0036] Li, F. , Y. Diao , J. Wang , et al. 2021. “Review of Cracked Tooth Syndrome: Etiology, Diagnosis, Management, and Prevention.” Pain Research and Management 2021: 1–12. 10.1155/2021/3788660 PMC8694987 34956432

[cre270323-bib-0037] Liao, W.‐C. , C.‐H. Chen , Y.‐H. Pan , M.‐C. Chang , and J.‐H. Jeng . 2021. “Vertical Root Fracture in Non‐Endodontically and Endodontically Treated Teeth: Current Understanding and Future Challenge.” Journal of Personalized Medicine 11, no. 12: 1375. 34945847 10.3390/jpm11121375 PMC8707645

[cre270323-bib-0038] Lim, M.‐J. , J.‐A. Kim , Y. Choi , C.‐U. Hong , and K.‐S. Min . 2017. “Differentiating Spontaneous Vertical Root Fracture in Endodontically Treated Tooth.” European Journal of Dentistry 11, no. 1: 122–125. 28435378 10.4103/ejd.ejd_160_16 PMC5379825

[cre270323-bib-0039] Lustig, J. P. , A. Tamse , and Z. Fuss . 2000. “Pattern of Bone Resorption in Vertically Fractured, Endodontically Treated Teeth.” Oral Surgery, Oral Medicine, Oral Pathology, Oral Radiology, and Endodontology 90, no. 2: 224–227. 10.1067/moe.2000.107445 10936842

[cre270323-bib-0040] Machoy, M. , J. Seeliger , L. Szyszka‐Sommerfeld , R. Koprowski , T. Gedrange , and K. Woźniak . 2017. “The Use of Optical Coherence Tomography in Dental Diagnostics: A State‐of‐the‐Art Review.” Journal of Healthcare Engineering 2017: 1–31. 10.1155/2017/7560645 PMC5534297 29065642

[cre270323-bib-0041] Mak, S. , and A. Thomas . 2022. “Steps for Conducting a Scoping Review.” Journal of Graduate Medical Education 14, no. 5: 565–567. 36274762 10.4300/JGME-D-22-00621.1 PMC9580325

[cre270323-bib-0042] Meister, F. , T. J. Lommel , and H. Gerstein . 1980. “Diagnosis and Possible Causes of Vertical Root Fractures.” Oral Surgery, Oral Medicine, Oral Pathology 49, no. 3: 243–253. 6928310 10.1016/0030-4220(80)90056-0

[cre270323-bib-0043] Minamino, T. , A. Mine , M. Matsumoto , et al. 2015. “Nondestructive Observation of Teeth Post Core‐Space Using Optical Coherence Tomography: Comparison With Microcomputed Tomography and Live Images.” Journal of Biomedical Optics 20, no. 10: 1. 10.1117/1.JBO.20.10.107001 26440617

[cre270323-bib-0044] Mireku, A. S. , E. Romberg , A. F. Fouad , and D. Arola . 2010. “Vertical Fracture of Root Filled Teeth Restored With Posts: The Effects of Patient Age and Dentine Thickness.” International Endodontic Journal 43, no. 3: 218–225. 20158533 10.1111/j.1365-2591.2009.01661.x PMC3353984

[cre270323-bib-0045] Moule, A. J. , and B. Kahler . 1999. “Diagnosis and Management of Teeth With Vertical Root Fractures.” Australian Dental Journal 44, no. 2: 75–87. 10452161 10.1111/j.1834-7819.1999.tb00205.x

[cre270323-bib-0046] Mullally, B. H. , and M. Ahmed . 2000. “Periodontal Signs and Symptoms Associated With Vertical Root Fracture.” Dental Update 27, no. 7: 356–360. 11218526 10.12968/denu.2000.27.7.356

[cre270323-bib-0047] Nakajima, Y. , Y. Shimada , M. Miyashin , Y. Takagi , J. Tagami , and Y. Sumi . 2012. “Noninvasive Cross‐Sectional Imaging of Incomplete Crown Fractures (Cracks) Using Swept‐Source Optical Coherence Tomography.” International Endodontic Journal 45, no. 10: 933–941. 22519809 10.1111/j.1365-2591.2012.02052.x

[cre270323-bib-0048] Nicopoulou‐Karayianni, K. , U. Bragger , and N. P. Lang . 1997. “Patterns of Periodontal Destruction Associated With Incomplete Root Fractures.” Dentomaxillofacial Radiology 26, no. 6: 321–326. 9482006 10.1038/sj.dmfr.4600264

[cre270323-bib-0049] de Oliveira, B. P. , A. C. Câmara , D. A. Duarte , et al. 2017. “Detection of Apical Root Cracks Using Spectral Domain and Swept‐Source Optical Coherence Tomography.” Journal of Endodontics 43, no. 7: 1148–1151. 28416309 10.1016/j.joen.2017.01.019

[cre270323-bib-0050] Pack, A. R. 1994. “A Report on Two Patients With Vertical Root Fracture: A Dilemma for the Periodontist, Endodontist, and Patient.” New Zealand Dental Journal 90, no. 401: 103–106. 7970332

[cre270323-bib-0051] Patel, S. , B. Bhuva , and R. Bose . 2022. “Present Status and Future Directions: Vertical Root Fractures in Root Filled Teeth.” International Endodontic Journal 55: 804–826. 35338655 10.1111/iej.13737 PMC9324143

[cre270323-bib-0052] Pinto, K. P. , A. F. A. Barbosa , E. J. N. L. Silva , A. P. P. Santos , and L. M. Sassone . 2023. “What Is the Microbial Profile in Persistent Endodontic Infections? A Scoping Review.” Journal of Endodontics 49, no. 7: 786–798.e7. 37211309 10.1016/j.joen.2023.05.010

[cre270323-bib-0053] PradeepKumar, A. R. , H. Shemesh , M. S. Nivedhitha , et al. 2021. “Diagnosis of Vertical Root Fractures by Cone‐Beam Computed Tomography in Root‐Filled Teeth With Confirmation by Direct Visualization: A Systematic Review and Meta‐Analysis.” Journal of Endodontics 47, no. 8: 1198–1214. 33984375 10.1016/j.joen.2021.04.022

[cre270323-bib-0054] Rathke, A. , H. Frehse , and B. Hrusa . 2022. “Vertical Root Fracture Resistance and Crack Formation of Root Canal‐Treated Teeth Restored With Different Post‐Luting Systems.” Odontology 110, no. 4: 719–725. 35523910 10.1007/s10266-022-00709-5 PMC9463252

[cre270323-bib-0055] Rivera, E. , and R. Walton . 2008. “Cracking the Cracked Tooth Code: Detection and Treatment of Various Longitudinal Tooth Fractures.” American Association of Endodontists Colleagues for Excellence Newsletter 2: 1–19.

[cre270323-bib-0056] Schneider, H. , K.‐J. Park , M. Häfer , et al. 2017. “Dental Applications of Optical Coherence Tomography (OCT) in Cariology.” Applied Sciences 7, no. 5: 472.

[cre270323-bib-0057] Schweitzer, J. L. , J. L. Gutmann , and R. Q. Bliss . 1989. “Odontiatrogenic Tooth Fracture.” International Endodontic Journal 22, no. 2: 64–74. 2689355 10.1111/j.1365-2591.1989.tb00508.x

[cre270323-bib-0058] Segarra, M. S. , Y. Shimada , A. Sadr , Y. Sumi , and J. Tagami . 2017. “Three‐Dimensional Analysis of Enamel Crack Behavior Using Optical Coherence Tomography.” Journal of Dental Research 96, no. 3: 308–314. 27872333 10.1177/0022034516680156

[cre270323-bib-0059] Sharma, R. , A. Trivedi , N. Singla , R. Kumar , and S. Arora . 2015. “Optical Coherence Tomography in Dentistry.” Journal of PEARLDENT 6, no. 1: 1–4.

[cre270323-bib-0060] Shemesh, H. , G. Van Soest , M.‐K. Wu , L. W. M. Van der Sluis , and P. R. Wesselink . 2007. “The Ability of Optical Coherence Tomography to Characterize the Root Canal Walls.” Journal of Endodontics 33, no. 11: 1369–1373. 17963966 10.1016/j.joen.2007.06.022

[cre270323-bib-0061] Shemesh, H. , G. van Soest , M.‐K. Wu , and P. R. Wesselink . 2008. “Diagnosis of Vertical Root Fractures With Optical Coherence Tomography.” Journal of Endodontics 34, no. 6: 739–742. 18498903 10.1016/j.joen.2008.03.013

[cre270323-bib-0062] Shimada, Y. , M. Yoshiyama , J. Tagami , and Y. Sumi . 2020. “Evaluation of Dental Caries, Tooth Crack, and Age‐Related Changes in Tooth Structure Using Optical Coherence Tomography.” Japanese Dental Science Review 56, no. 1: 109–118. 33033549 10.1016/j.jdsr.2020.08.001 PMC7533308

[cre270323-bib-0063] Suassuna, F. C. M. , A. M. A. Maia , D. P. Melo , A. C. D. Antonino , A. S. L. Gomes , and P. M. Bento . 2018. “Comparison of Microtomography and Optical Coherence Tomography on Apical Endodontic Filling Analysis.” Dentomaxillofacial Radiology 47, no. 2: 20170174. 29106310 10.1259/dmfr.20170174 PMC5965905

[cre270323-bib-0064] Sugaya, T. , M. Kawanami , H. Noguchi , H. Kato , and N. Masaka . 2001. “Periodontal Healing After Bonding Treatment of Vertical Root Fracture.” Dental Traumatology 17, no. 4: 174–179. 11585144 10.1034/j.1600-9657.2001.170407.x

[cre270323-bib-0065] Talwar, S. , S. Utneja , R. R. Nawal , A. Kaushik , D. Srivastava , and S. S. Oberoy . 2016. “Role of Cone‐Beam Computed Tomography in Diagnosis of Vertical Root Fractures: A Systematic Review and Meta‐Analysis.” Journal of Endodontics 42, no. 1: 12–24. 26699923 10.1016/j.joen.2015.09.012

[cre270323-bib-0066] Tamse, A. , Z. Fuss , J. Lustig , Y. Ganor , and I. Kaffe . 1999. “Radiographic Features of Vertically Fractured, Endodontically Treated Maxillary Premolars.” Oral Surgery, Oral Medicine, Oral Pathology, Oral Radiology, and Endodontology 88, no. 3: 348–352. 10.1016/s1079-2104(99)70041-7 10503867

[cre270323-bib-0067] Torabinejad, M. , A. F. Fouad , and S. Shabahang . 2020. *Endodontics E‐Book* . Elsevier Health Sciences.

[cre270323-bib-0068] Tricco, A. C. , E. Lillie , W. Zarin , et al. 2018. “PRISMA extension for Scoping Reviews (PRISMA‐ScR): Checklist and Explanation.” Annals of Internal Medicine 169, no. 7: 467–473. 30178033 10.7326/M18-0850

[cre270323-bib-0069] Tsesis, I. , E. Rosen , A. Tamse , S. Taschieri , and A. Kfir . 2010. “Diagnosis of Vertical Root Fractures in Endodontically Treated Teeth Based on Clinical and Radiographic Indices: A Systematic Review.” Journal of Endodontics 36, no. 9: 1455–1458. 20728708 10.1016/j.joen.2010.05.003

[cre270323-bib-0070] Vadivambal, R. , and D. S. Jayas . 2015. *Bio‐Imaging: Principles, Techniques, and Applications* . CRC Press.

[cre270323-bib-0071] Walton, R. E. 2017. “Vertical Root Fracture.” Journal of the American Dental Association 148, no. 2: 100–105. 28129797 10.1016/j.adaj.2016.11.014

[cre270323-bib-0072] Wilcox, L. R. , C. Roskelley , and T. Sutton . 1997. “The Relationship of Root Canal Enlargement to Finger‐Spreader Induced Vertical Root Fracture.” Journal of Endodontics 23, no. 8: 533–534. 9587326 10.1016/S0099-2399(97)80316-0

[cre270323-bib-0073] Wilder‐Smith, P. , L. Otis , J. Zhang , and Z. Chen . 2008. “Dental OCT.” In *Optical Coherence Tomography: Technology and Applications* , 1151–1182. Springer.

[cre270323-bib-0074] Ţogoe, M.‐M. , E.‐L. Crăciunescu , F.‐I. Topală , et al. 2021. “Endodontic Fillings Evaluated Using En Face OCT, MicrOCT and SEM.” Romanian Journal of Morphology and Embryology 62, no. 3: 793–800. 35263408 10.47162/RJME.62.3.17 PMC9019634

